# Prognostic factors for functional recovery after lingual nerve reconstruction using an artificial nerve conduit

**DOI:** 10.1186/s40902-026-00500-4

**Published:** 2026-01-14

**Authors:** Shigeyuki Fujita, Shigeru Suzuki, Osamu Sakaguchi, Itaru Tojyo

**Affiliations:** 1 North Osaka Housennka Hospital, Ibaragi-city Osaka, Japan; 2 Wakayama Medical University, Wakayama City, Japan; 3 Oral Medicine, Kyushu Dental University, Fukuoka, Japan

## Abstract

**Background:**

Lingual nerve injury following dental procedures, such as lower third molar extractions, can cause significant sensory deficits. For patients with persistent severe symptoms, surgical reconstruction using a nerve conduit is often considered. However, the degree of recovery varies, and the optimal timing of intervention and the significance of the nerve gap distance remain subjects of clinical debate. Objectives: Using the Medical Research Council Scale (MRCS) as a standardized measure of sensory function, this study aims to determine the independent effects of the timing of surgery, nerve gap length, and other potential prognostic factors on nerve functional recovery, specifically defining success as MRCS S3+ or higher. Methods: This study retrospectively analyzed a cohort of 49 patients who underwent lingual nerve repair surgery. The success of nerve recovery was evaluated using two established criteria: the standard Functional Sensory Recovery (FSR), MRCS S3 or higher, and the strict MRCS S3+ or higher criteria based on American Society of Plastic Surgeons (ASPS) criteria. The MRCS S3+ or higher criteria was designated as the primary outcome for all multivariate analyses. The time to surgery variable was logarithmically transformed, Log (Time to Surgery, months), to account for the highly skewed distribution. Statistical analysis used univariate and multivariate logistic regression to assess the association between each predictor and postoperative MRCS score. A secondary analysis examined predictors for allodynia resolution.

**Results:**

The logarithmically transformed time to surgery, Log (Time to Surgery, months), was the sole statistically significant independent predictor for achieving MRCS S3+ (Odds Ratio OR = 0.236, 95% CI: 0.063—0.887, *P* = 0.032). This indicates that earlier intervention significantly increases the odds of functional recovery. Nerve gap length was not a significant predictor (OR = 0.941, *P* = 0.518). Furthermore, no variable was found to be a significant predictor for allodynia resolution (*P* > 0.05).

**Conclusion:**

Earlier surgical intervention, quantified by Log (Time to Surgery), is an independent and critical factor for achieving MRCS S3+ functional sensory recovery after lingual nerve repair. The distance of the nerve gap did not show an independent predictive effect on the final sensory outcome.

## Background

The lingual nerve (LN) is susceptible to iatrogenic injury, most commonly during the extraction of lower third molars [1–3]. This damage frequently leads to severe sensory deficits, including dysesthesia, hypesthesia, and pain such as allodynia, significantly impacting patients' quality of life. Although conservative treatment is the initial approach, patients with persistent severe symptoms often require microsurgical nerve repair. For peripheral nerve injuries, the gold standard for management is microsurgical repair, which often involves the placement of a nerve conduit or autograft when a gap exists. While several factors influence the success of nerve regeneration, two key elements remain subjects of ongoing debate in clinical practice: the timing of the surgery and the length of LN gap. The detrimental effect of delayed intervention is well-established in general peripheral nerve literature; however, the specific cutoff timing and its independent predictive value in LN repair remains unclear [4–6]. Furthermore, although longer nerve gaps are theoretically associated with poorer outcomes due to increased regeneration distance, this relationship is not always statistically confirmed in clinical studies involving LN or other cranial nerves. The definition of a truly successful outcome in nerve repair also lacks consensus, with the standard FSR, MRCS S3 or better often failing to reflect a clinically meaningful, high-quality recovery. This study retrospectively analyzes a cohort of patients who underwent LN repair surgery, designating the strict standard of MRCS S3+ or higher as the primary outcome for functional sensory recovery. This approach allows us to use multivariate logistic regression to rigorously assess the critical influence of the logarithmically transformed time to surgery and nerve gap distance, while controlling for other patient and injury characteristics.

## Methods

### Patients

**Fig. 1:**
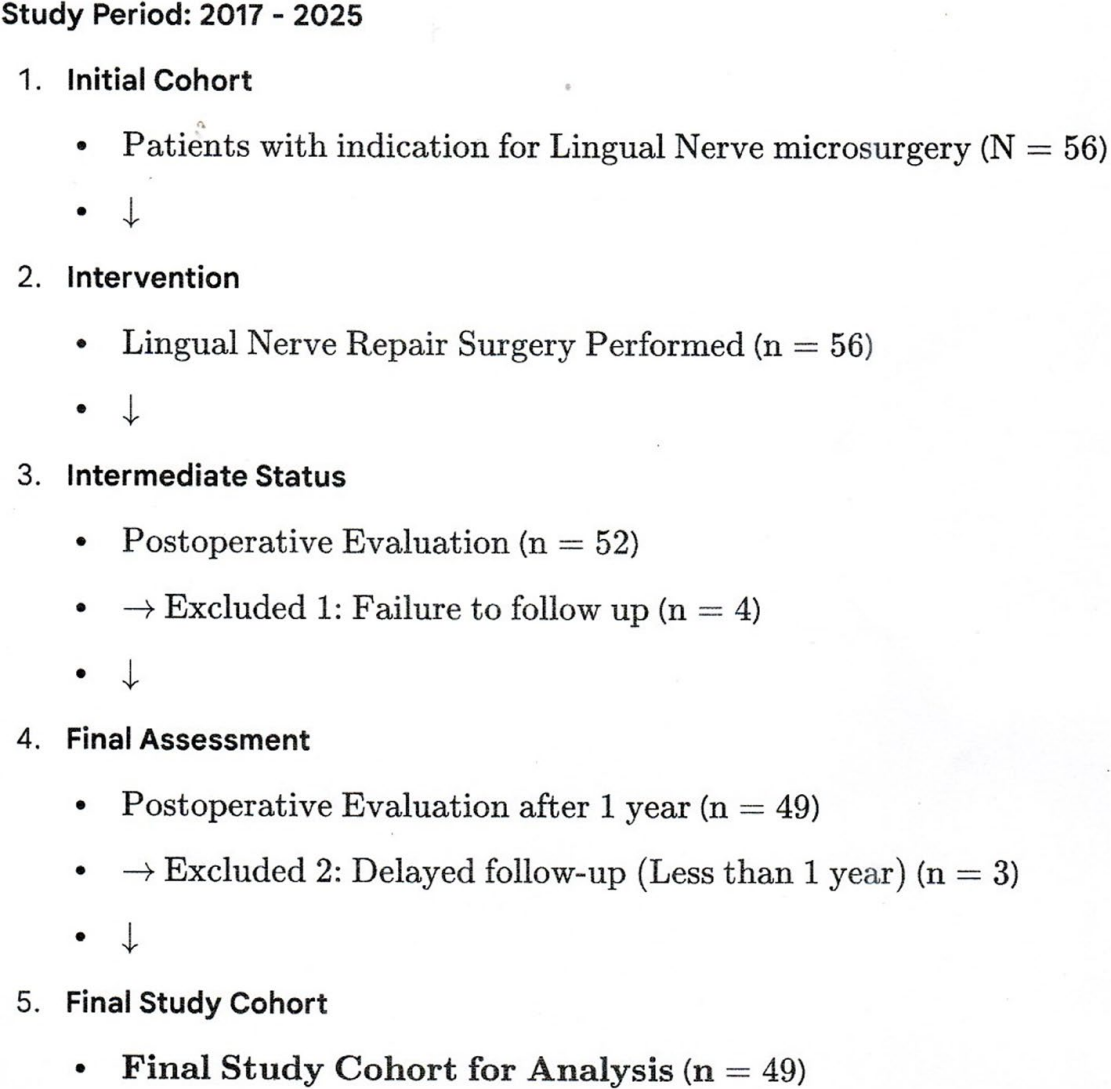
Patient Selection flow chart flow chart detailing the inclusion and exclusion criteria, resulting in the final study cohort of 49 patients

This retrospective study included patients who underwent LN repair surgery at the　Department of Oral and Maxillofacial Surgery between 2017 and 2025. Inclusion criteria were　patients with an iatrogenic LN injury undergoing primary surgical repair using a nerve conduit or autograft. Patients with follow-up periods less than 1 year or those lost to follow-up were excluded. The final study cohort comprised 49 patients (Fig. 1). Surgical Technique: All surgeries were performed by a single surgeon using a standardized technique. A curvilinear incision was made in the floor of the mouth, and the LN was identified and mobilized. The injured segment was resected until healthy nerve tissue was confirmed at both proximal and distal stumps. The resulting nerve gap was measured directly. The nerve was repaired using a nerve conduit (Renerve®) in all 49 cases, following resection of the injured segment [7]. Data Collection: Clinical data collected included patient demographics (age, gender), injury characteristics (time to surgery in months, nerve gap in mm), surgical details (operation time in minutes), and medical history (diabetes mellitus, psychotropic drug use). Time to surgery was defined as the interval between the date of injury and the date of nerve repair. Outcome Assessment: Sensory function was assessed pre- and postoperatively at least one year after surgery using MRCS for sensory function, ranging from S0 (no sensory recovery) to S4 (complete recovery). Recovery success was evaluated using two established criteria: the standard FSR defined as MRCS S3 or higher criteria, and the strict good recovery defined as MRCS S3+ or higher ASPS criteria. MRCS S3+ or higher criteria was designated as the primary outcome for all subsequent multivariate analyses. A secondary outcome was the resolution of allodynia, defined as the disappearance of preoperative allodynia at the final follow-up. Statistical Analysis: Descriptive statistics were summarized using median (interquartile range, IQR) for continuous variables and number (percentage, N %) for categorical variables. Due to the highly skewed distribution of the "Time to Surgery" variable, it was logarithmically transformed, Log (Time to Surgery, months), for all regression analysis (Table 1). Univariate logistic regression analysis was performed to identify factors associated with the primary outcome (MRCS S3+ recovery) (Table 2). Variables with a P-value of less than 0.20 in the univariate analysis was included in the multivariate logistic regression model to identify independent predictors for MRCS S3+ recovery (Table 3.). Finally, a multivariate logistic regression model was used to identify independent predictors for allodynia resolution (Table 4). Statistical significance was set at *P* < 0.05. All analyses were performed using JMP Pro 16.0 (SAS Institute, Cary, NC, USA).

**Table 1:** Patient demographics, injury characteristics, and outcomes (*n* = 49) Summary of key patient characteristics, surgical factors, and primary outcomes. (Includes age, gender, time to surgery, nerve gap, operation time, preoperative MRCS, Postoperative MRCS, Allodynia Pre/Post)

Characteristic			Data Range		Statistical Value(Mean ± SD)
			(Min–Max)		{Median(IQR)] or n(%)
Age(years)			18–63		35.5 ± 11.2 [33.0(27.0–42.0)]
Gender			Male:10/Female:39		Male 10 (20.4%)/Female 39 (79.6%)
Time to Surgery (months)			3–204		13.6 ± 28.7 [6.0(5.0–12.0)]
Nerve Gap (mm)			10‐25		15.6 ± 3.3 [15.0(13.0–18.0)]
Operation Time			201–467		311.1 ± 75.3 [281.0(262.0–375.0)]
Preoperative Allodynia (Yes)			*n* = 23		23(46.9%)
(*N* = 49 among total)					
Comorbidities: Diabetes Mellitus (Yes)			*n* = 2		2 (4.1%)
Comorbidities: Psychotropic Drug Use (Yes)			*n* = 11		11 (22.4%)
Postoperative MRCS ≥ S3+			*n* = 43		43 (87.8%)
(*N* = 49 among total)					
Allodynia Resolution			*n* = 20		20 (40.8%)
(*N* = 49 among total)					
Preoperative MRCS distribution					
MRCS	S0 ~ S2	S2+	S3	S3+	S4
n	0	17	31	1	0
%	0	34.7	63.2	2	0
Postoperative MRCS distribution					
MRCS	S0 ~ S2	S2+	S3	S3+	S4
n	0	0	12	31	6
%	0	0	24.4	63.2	12.2

MRCS: Medical Reseach Council Scale. The success criteria in this study is strictly defined as an MRCS score of S3+ or higher, reflecting the standard for good functional recovery in peripheral nerve surgery

Allodynia Resolution reffers o the disappearance of allodynia at the final follow-up, reported as the percentage of the total patient cohort (*N* = 49)

Note that 23 patients who presented with preoperative S3+ and S3 reported allodynia, but 20 achieved resolution of this symptom postoperatively

**Table 2:** Univariate logistic regression analysis for MRCS S3+ Recovery (n = 49) Univariate analysis showing the predictors associated with achieving functional sensory recovery, strictly defined as MRCS S3+ or higher

Characteristic	OR (Unadjusted)	95% CI	P-value
Log (Time to Surgery, months)	0.223	[0.063,0.795]	0.021
Nerve Gap (mm)	0.933	[0.793, 1.096]	0.395
Age (years)	0.985	[0.937,1.036]	0.563
Gender (Male vs Female)	1.294	[0.334,5.013]	0.707
Diabetes Mellitus (Yes vs No)	1.765	[0.311,10.01]	0.518﻿
Preoperative Allodynia (Yes vs No)	0.725	[0.187,2.809]	0.640
Preoperative FSR (Yes vs No)	0.725	[0.187,2.809]	0.640
Psychotropic drug use (Yes vs No)	0.625	[0.101,3.864]	0.612

The outcome is achieving MRCS 53+ or better at the final follow-up (1 year or more)

. OR (Unadjusted): Simple Odds Ratio for each variable predicting MRCS 53+ recovery

. Model Selection Rationale: Factors with a P-value < 0.20 (e.g., Log (Time to Surgeryl) were considered for the subsequent multivariable anallysis

**Table 3:** Multivariate logistic regression analysis for functional sensory recovery (MRCS S3+) (n = 49) Multivariate analysis showing the independent predictors for achieving functional sensory recovery (MRCS S3+ or higher)

Characteristic	OR (Adjusted)	95% CI	P-value
Log (Time to Surgery, months)	0.236	[0.063,0.887]	0.032
Nerve Gap (mm)	0.941	[0.787,1.125]	0.518
Preoperative Allodynia (Yes vs No)	0.569	[0.111,2.915]	0.499
Psychotropic drug use (Yes vs No)	0.481	[0.065, 3.565]	0.457

The outcome is achieving MRCS S3-+ or better at the final follow-up (1 year or more)

· The final model was adjusted for variables with P < 0.20 in the univariate analysis (Table 2) plus clinically relevant variables

· Interpretation: Log (Time to Surgery) was the only statistically significant independent predictor for achieving MRCS S3+ recovery. For every unit increase in Log (Time to Surgery), the odds of MRCS S3+ recovery decrease by 76.4% (1 - 0.236)

**Table 4:** Multivariate logistic regression analysis for allodynia resolution (n = 49) Multivariate analysis showing the independent predictors for the resolution of preoperative allodynia at the 1 year follow up

Characteristic	OR (Adjusted)	95% CI	P-value
Preoperative Allodynia (Yes vs No)	2.312	[0.511, 10.45]	0.276
Preoperative Drugs (Yes vs No)	0.801	[0.205, 3.125]	0.751
Gender (Male vs Female)	1.092	[0.271,4.385]	0.899
Log (Time to Surgery, months)	1.215	[0.370,3.991]	0.751

The outcome is Allodymia Resolution at the final follow-up (1 year or more)

. The final model was adjusted for all potential predictors based on clinical and statistical criteria

. Interpretation: No variable was found to be a statistically significant independent predictor for Allodynia Resolution

## Results

### Patient characteristics and outcomes

A total of 49 patients were included in the final analysis (Fig. 1). Patient characteristics, injury features, and outcomes are detailed in Table 1. The median age of the cohort was 33 years (IQR: 27.0—42.0 years), and 79.6% (*n* = 39) were male. The median nerve gap was 15 mm (IQR: 13.0–18.0 mm). The median time from injury to surgery was 6.0 months (IQR: 5.0—12.0 months). The MRCS S3+ recovery success rate was 87.8% *n* = 43/49. Preoperative allodynia was observed in 46.9% (*n* = 23/49) of patients, and of those, 86.9% (*n* = 20/23) experienced resolution of allodynia after surgery. Predictors for MRCS S3+ Recovery: The univariate logistic regression analysis showed that Log (Time to Surgery, months) was the only variable significantly associated with MRCS S3+ recovery (OR = 0.223, 95% CI: 0.063—0.795, *P* = 0.021). Nerve gap, psychotropic drug use, and other factors was not statistically significant predictors in the univariate analysis (Table 2). The multivariate logistic regression model included Log (Time to Surgery), Nerve Gap, Preoperative Allodynia, and Preoperative Psychotropic drug use. Log (Time to Surgery, months) remained the sole statistically significant independent predictor for MRCS S3+ recovery (OR = 0.236, 95% CI: 0.063—0.887, *P* = 0.032). For every unit increase in the logarithm of time to surgery, the odds of achieving MRCS S3+ decreased by approximately 76%. Nerve gap was not an independent predictor (OR = 0.941, *P* = 0.518) (Table 3.). Predictors for Allodynia Resolution: Multivariate logistic regression analysis for allodynia resolution included Log (Time to Surgery), Preoperative Allodynia, Preoperative Psychotropic drug use, Gender. No variable was found to be a statistically significant independent predictor for the resolution of allodynia (*P* > 0.20) (Table 4).

## Discussion

### Clinical significance and strategies of early intervention

Definition of Early Intervention: Based on the definitive results of multivariate analysis (time to surgery was the only significant prognostic factor, *P* = 0.032), "early intervention" in clinical practice would be defined as a repeat diagnosis using Clinical Neurosensory Testing (CNT), within 3–6 months after injury that shows no signs of improvement, a positive Tinel's sign, or persistent allodynia, suggesting the need for prompt referral to an oral surgeon [8–11]. The Critical Distinction Between FSR (S3) and good Recovery (S3+): The definition of FSR is defined as recovery to S3 or better, primarily addressing whether a basic level of protective sensation has been achieved (recovery of S3: Sensation with incorrect localization, allowing for object manipulation) [12, 13]. In contrast, ASPS and general peripheral sensory nerve reconstruction literature often employs a more stringent standard, classifying S3+ as good (Sensation with correct localization, but residual two-point discrimination deficit) or S4 as Excellent recovery, while S3 and below are classified as Poor [14]. This study utilized both criteria-FSR (MRCS S3 or better) and ASPS's more rigorous standard (MRCS S3+ or better) -to evaluate recovery and compare the impact of prognostic factors. The two criteria are fundamentally different: FSR focuses on the *existence* of function, while ASPS focuses on the *quality* of function. Our multivariate analysis demonstrated that the time-to-surgery variable became statistically significant only when using the more rigorous S3+ threshold as the primary outcome. This finding strongly validates the use of the S3+ criterion, suggesting that MRCS S3-while meeting the technical definition of FSR-should not be considered a truly successful or good clinical outcome in LN repair.

### MR Neurography (MRN) and the study period context

While MR Neurography (MRN) provides valuable morphological data, recent studies have demonstrated a statistically significant correlation between MRN-derived nerve gap length and final functional sensory outcomes [15]. However, it is essential to note that the high-resolution MRN necessary for precise LN visualization and accurate nerve gap measurement was not routinely available or standardized in our general clinical setting during the 2017–2025 study period. Consequently, our prognostic analysis relied on the universally accepted gold-standard of CNT using the MRCS scale as the most reliable, practical, and accessible indicator of functional deficit and recovery progression. Furthermore, while correlation studies exist in other domains (e.g., digital nerve repair, which often reports high MRN specificity), direct comparisons with LN injury are difficult due to fundamental anatomical, functional, and imaging field differences in the oral cavity. This field-specific constraint reinforces our decision to prioritize the clinically universal MRCS scores for establishing prognostic factors.

### Timing of surgery and prognosis

Statistically, multivariate analysis including logarithmic transformation of time to surgery showed a significant association with achieving a useful recovery (MRCS S3+). Therefore, early intervention maximizes the likelihood of achieving S3+. However, even with delayed surgery, some degree of functional improvement remains possible, which may have a chance of recovery. Supporting this, our individual case studies have shown good functional recovery even in patients who waited very long (e.g., 8 or 17 years) before surgery [16]. Therefore, preoperatively informing patients that surgery will likely result in a poor prognosis or is contraindicated due to the long time since injury should be avoided. This is consistent with Robinson's view that LN repair should be considered regardless of the length of time since LN injury [17].

### Limits of nerve gap length and nerve diameter

The debate surrounding the maximum length of the nerve gap that can be bridged without requiring autologous nerve grafting is critical. Perrelle suggested that 15 mm is the limit for using a nerve conduit alone [18]. However, this guideline may depend significantly on the nerve's diameter. Numerous animal experiments involving the repair of thinner nerves (such as the sciatic nerves of small animals like rats and rabbits) have demonstrated nerve regeneration of up to 30 mm using artificial conduits alone [19, 20]. In contrast, experiments using large animals (e.g., sheep and pigs) with thicker nerves, such as the median nerve, failed to observe nerve regeneration when bridging gaps of 8 cm using only a conduit [21, 22]. This discrepancy supports the theory that the diameter of the nerve significantly restricts regenerative capacity. The concentration of neurotrophic factors within the conduit are essential for advancing nerve regeneration, and there is a physical limit to this capacity. As defined by the regenerative field (V), the maximum regenerative distance (L) will inevitably be shorter for nerves with a larger diameter: V = πr^2^X L, where r is the nerve radius [23, 24]. If the radius (r) is doubled, the potential regenerative distance (L) is reduced to one-fourth the original length. While nerve grafting techniques using cell suspensions (e.g., Schwann cells) show promising results in animals for small defect sizes over 30 mm, the translation to human organisms is limited by highly regulated laws for the transplantation of human stem cells [25]. Because the human LN is relatively thin, measuring approximately 3 mm in diameter, successful regeneration using nerve conduits alone is highly likely for defects up to 30 mm in length. We have observed a case in which a 25 mm nerve defect was left untreated for six years, and the patient experienced good functional recovery. Clinically, we believe that complete radical resection of the damaged and degenerated nerve observed within the field of view during surgery, to the point where normal nerve stumps can be confirmed, and the procedure of ensuring an environment suitable for regeneration, is a fundamental yet important technique that can eliminate allodynia. If an optimal environment for regeneration is reliably established after complete resection of the damaged nerve, a favorable prognosis may be achieved even if the resection distance slightly exceeds conventional limits. This point remains highly controversial and requires further investigation in a larger number of cases.

### Timing vs. nerve gap length

Our results strongly support the biological principle that delayed repair negatively impacts the success of nerve regeneration. With the passage of time, the regenerative capacity of the distal nerve stump and the integrity of the endoneurium deteriorate, leading to poorer outcomes. The logarithmic transformation of the time variable is crucial as it accurately models the clinically observed non-linear relationship: the rate of decline in recovery potential is rapid in the early months and then gradually plateaus, underscoring the urgency of early intervention. A key finding is the lack of independent prognostic value for nerve gap length in predicting MRCS S3+ recovery (*P* = 0.518). While basic science and animal models demonstrate that longer gap present a significant obstacle to successful regeneration, this relationship appears to be obscured by the dominant influence of the time factor in the clinical setting. Specifically, studies using long-segment nerve grafts in large animal models (e.g., sheep, swine, rabbits) have shown that regeneration becomes increasingly limited beyond a certain critical gap length (e.g., 6 cm in some models), often due to increased Schwann cell senescence and depletion of neurotrophic factors in the distal stumps [26]. Our finding suggests that for the typical range of LN gaps observed in clinical practice (median 15 mm), the negative effect of a delayed surgery appears to outweigh the theoretical negative effect of the nerve gap distance. This is a critical finding for clinical decision-making, suggesting that microsurgery should not be postponed solely due to concerns about the length of the defect, but rather accelerated regardless of the gap size to mitigate the damage caused by chronicity. Furthermore, our study found no significant independent predictors for the resolution of allodynia. While surgery resulted in the resolution of allodynia in 86.9% of affected patients. In the three cases in which mild allodynia persisted after surgery, common characteristics of diabetes or preoperative psychotropic drug use was observed. This observation is very important in preoperative patient counseling, and suggests that patients with these systemic diseases should be explained the possibility that they may be at risk of not completely curing their neuropathic pain symptoms, even after technically successful nerve repair. the multifactorial nature of neuropathic pain means that its resolution may depend on factors (e.g., patient compliance, pain medication management, psychological state) not fully captured by the anatomical and timing variables included in our model.

### Limitations

This study has several limitations inherent to its retrospective design, including the possibility of selection bias and confounding factors not included in the model. Furthermore, all surgeries were performed by a single surgeon, which promotes standardization but limits the generalizability of findings to different surgical techniques. Furthermore, the relatively small sample size (*n* = 49) may have limited statistical power, as it was not possible to confirm the significance of clinical factors (e.g., nerve defect distance) and may have affected the wide confidence intervals.

## Conclusions

In this retrospective analysis, ordinal scaling and multivariate logistic regression analysis revealed a strong and significant association between early surgical intervention and favorable outcome, defined as an MRCS score of S3+ or higher after LN repair. Log-transformed time from injury to surgery was the only statistically significant independent predictor of achieving functional sensory recovery of MRCS S3+ or higher after LN repair. This study suggests the need for early referral and prompt surgical intervention for patients with LN injury, regardless of the measured nerve gap distance. Furthermore, this analysis revealed that surgical intervention provides significant clinical benefit in alleviating preoperative allodynia, an important factor determining patient quality of life. In contrast, other factors, such as operative time, age, gender, and diabetes status, did not show significant trends in either analysis. These findings emphasize the dual importance of early surgical repair and accurate anatomical knowledge to optimize postoperative sensory recovery and pain symptom relief in patients with LN injury. Future prospective, multicenter studies with larger patient numbers are needed to further elucidate the complex prognostic factors that influence nerve regeneration and pain management.

## Data Availability

Please contact the author for data requests.

## Abbreviations

LN: Lingual nerve
MRCS: Medical Research Council Scale
FSR: Functional Sensory Recovery
CNT: Clinical Neurosensory Testing
ASPS: American Society of Plastic Surgeons
MRN: MR Neurography
